# Relation between chronic rhinosinusitis and gastroesophageal reflux in adults: systematic review^☆^

**DOI:** 10.1016/j.bjorl.2016.05.012

**Published:** 2016-07-14

**Authors:** Guilherme Constante Preis Sella, Edwin Tamashiro, Wilma Terezinha Anselmo-Lima, Fabiana Cardoso Pereira Valera

**Affiliations:** Universidade de São Paulo, Faculdade de Medicina de Ribeirão Preto, Departamento de Oftalmologia, Otorrinolaringologia e Cirurgia de Cabeça e Pescoço, Ribeirão Preto, SP, Brazil

**Keywords:** Rhinosinusitis, Laryngopharyngeal reflux, Gastroesophageal reflux disease, pH-metry, Proton pump inhibitor, Rinossinusite, Refluxo laringofaríngeo, Doença do refluxo gastroesofágico, pHmetria, Inibidor de bomba de prótons

## Abstract

**Introduction:**

The relationship between gastroesophageal reflux disease (GERD) and chronic rhinosinusitis (CRS) is still a controversial issue in literature.

**Objective:**

A systematic review of the association between these two diseases in adult patients.

**Methods:**

Systematic review in PubMed and Cochrane Database with articles published between 1951 and 2015. We included all articles that specifically studied the relationship between CRS and GERD.

**Results:**

Of the 436 articles found, only 12 met the inclusion criteria. Eight cross-sectional articles suggest a relation between CRS and GERD, especially on CRS that is refractory to clinical or surgical treatment. However, the groups are small and methodologies are different. Four other longitudinal studies have assessed the effect of treatment with proton pump inhibitors (PPIs) on the improvement of symptoms of CRS, but the results were conflicting.

**Conclusions:**

There seems to be relative prevalence of reflux with intractable CRS. There is still a lack of controlled studies with a significant number of patients to confirm this hypothesis. Few studies specifically assess the impact of treatment of reflux on symptom improvement in patients with CRS.

## Resumo

**Introdução:**

A relação entre a Doença do Refluxo Gastroesofágico (DRGE) e a Rinossinusite Crônica (RSC) ainda é tema de controvérsia em literatura.

**Objetivo:**

Revisão sistemática sobre a associação entre essas duas doenças em pacientes adultos.

**Método:**

Revisão sistemática no Pubmed e Cochrane Database com os artigos publicados entre 1951 e 2015. Foram incluídos todos os artigos que estudassem especificamente a relação entre RSC e DRGE.

**Resultados:**

Dos 436 artigos encontrados, apenas 12 satisfaziam os critérios de inclusão. Oito artigos transversais sugerem relação da RSC com a DRGE, especialmente sobre a RSC refratária a tratamento clínico ou cirúrgico prévio. No entanto, os grupos são pequenos e as metodologias são muito diferentes. Outros quatro estudos longitudinais avaliaram o efeito do tratamento com Inibidores de Bomba de Prótons (IBP) sobre a melhora dos sintomas de RSC, porém os resultados foram discordantes.

**Conclusões:**

Parece haver relação de prevalência de refluxo e RSC de difícil controle. Ainda faltam estudos controlados com um número expressivo de pacientes para que se confirme essa hipótese. São escassos os estudos que avaliem especificamente o impacto do tratamento de refluxo na melhora dos sintomas em pacientes com RSC.

## Introduction

Chronic rhinosinusitis (CRS) remains a major problem of public health worldwide.^1^ The broad consensus for recommended treatment is based on an optimal medical treatment emphasizing the use of corticosteroids.^2^, ^3^ Endoscopic nasal surgery (ENS) is indicated for cases that fail maximum medical treatment. However, multiple factors have been implicated as reasons that cases of CRS become refractory after optimized treatment including genotypic or phenotypic alteration of the mucosa, scars and synechiae, allergies, smoking and gastroesophageal acid reflux.^4^, ^5^

Especially in children many studies have postulated a relationship between CRS and acid reflux, both gastroesophageal reflux disease (GERD) and laryngopharyngeal reflux (LPR).^6^, ^7^, ^8^ However, it is difficult to establish a direct relationship between CRS and GERD, since both entities are highly prevalent, which makes it easier for them to coexist independently.^9^ In addition, to date there is no documented evidence of this possible relationship in adults.

Some theories of a relation between acid reflux and CRS were raised. The first is the direct exposure of the nasal and nasopharyngeal mucosa to gastric acid, causing inflammation of the mucosa and impaired mucociliary clearance, which could cause obstruction of sinus ostia and recurrent infections.^10^, ^11^ It is known that pH variations affect ciliary motility and morphology in the respiratory mucosa.^12^

The second hypothesis is a relationship mediated by the vagus nerve, a mechanism already proven in the lower airway^13^, ^14^ and in the nasal mucosa of patients with rhinitis,^10^ but not in patients with CRS. Dysfunction of the autonomic nervous system can lead to reflex sinonasal swelling and inflammation, and consequent blockage of the ostia. Wong et al.^15^ demonstrated that by infusing saline with hydrochloric acid in the lower esophagus of healthy volunteers, there was increased production of nasal mucus, increased score of nasal symptoms, and reduced peak nasal inspiratory flow, which would support this theory.

A final mechanism would be the direct role of *Helicobacter pylori* (*H. pylori*). Koc et al.^16^ observed *H. pylori* present in nasal polyps, but not in control tissues, whereas Morinaka et al.^17^ found *H. pylori* through polymerase chain reaction (PCR) in the nasal mucosa of patients who have CRS and gastroesophageal reflux complaints. However there are conflicting findings in the literature as to whether there is a greater frequency of *H. pylori* in the nasal mucosa of patients with CRS.^18^

More importantly, previous review studies failed to show a clear evidence-based relationship between CRS and GERD^19^, ^20^ in adults. Since these reviews were published at least four years ago, our objective was a new systematic review on the topic, to gather all the current evidence published around this issue, and to evaluate the quality and relevance of the interaction between GERD and CRS in adults.

## Methods

For the implementation of this systematic review, we selected all the items present in the PubMed library, developed by the National Center for Biotechnology Information (NCBI) of the US National Library of Medicine (NLM) (www.ncbi.nlm.nih.gov/PubMed), and in the library of the Cochrane Database (http://www.cochrane.org). The word search strategies were: Gastroesophageal reflux; OR GERD; OR GORD; OR laryngopharyngeal reflux; OR nasopharyngeal reflux; OR pH-metry. Associated with: sinusitis; OR chronic rhinosinusitis; OR chronic sinusitis; OR CRS; OR post-nasal drip. The minimum requirements for the selection were articles in English, which had an abstract, published between January 1, 1950 and December 31, 2015.

The final search resulted in 436 articles. Of these, 415 had abstracts in their respective databases, and 38 were excluded because they were not in English.

The abstracts of the selected articles were carefully read by two authors. After reading, only those articles that specifically evaluated the relationship between chronic rhinosinusitis and acid reflux in adults were included, resulting in 12 articles.

For this study, CRS criteria were used, according to the latest version of EPOS 2012,^2^ being defined as inflammation of the nose and paranasal sinuses, characterized by two or more symptoms associated with endoscopic or tomographic signs existing more than 12 weeks (Table 1). The articles considered were those about both forms of CRS, with or without nasal polyposis (NSP) for the research in question.

**Table 1:** Definition of chronic rhinosinusitis in adults, according to EPOS 2012.^2^

Symptoms	Nasal blockage/obstruction
	Nasal congestion or rhinorrhea (anterior/posterior nasal drip):
	- ±facial pain/pressure
	- ±Smell reduction or loss

Endoscopic signs	Nasal polyps, and/or
	Mucopurulent discharge, mainly from middle meatus, and/or
	Mucosa obstruction/edema mainly on middle meatus

and/or CT changes	Changes on the mucosa within ostiomeatal complex, and/or paranasal sinuses

To include the diagnosis of GERD, the articles considered were those whose patients had typical symptoms such as: heartburn and regurgitation, especially at night, the presence of lesions in esophagus mucosa at endoscopy, and changes in 24-h ambulatory pH-metry.^21^ This latter test is considered the gold standard for diagnosis of GERD by these researchers.

Patients were considered as having LPR if they showed extraesophageal symptoms such as mucus, dysphonia and cough, preferably at daytime, and other subjective symptoms such as globus sensation, excessive mucus and postnasal drip,^22^ as well as positivity in at least one of these scores: Reflux Symptom Index (RSI) (Table 2),^23^ or Reflux Finding Score (RFS),^24^ translated into Portuguese as *Escala de Achados Endolaríngeos de Refluxo* (Table 3).^25^

**Table 2:** Laryngopharyngeal Reflux Symptom Index (RSI).^23^ A RSI > 13 can be indicative of laryngopharyngeal reflux.

Last month, how these problems affected you?	0 = No problem					
	5 = important problem					
1. Hoarseness or voice problem	0	1	2	3	4	5
2. Clearing the throat	0	1	2	3	4	5
3. Excessive throat or nose secretions	0	1	2	3	4	5
4. Difficulty swallowing food, liquids or tablets	0	1	2	3	4	5
5. Cough after eating or after lying down	0	1	2	3	4	5
6. Difficulties breathing or choking episodes	0	1	2	3	4	5
7. Excessive cough	0	1	2	3	4	5
8. Sensation of something sticking on the throat	0	1	2	3	4	5
9. Heartburn, chest pain, indigestion or stomach acid in the mouth	0	1	2	3	4	5

**Table 3:** Endolaryngeal reflux findings score (RFS).^24^, ^25^ A RFS > 11 in the appropriate clinical situation is strongly suggestive of laryngopharyngeal reflux.

Endolaryngeal reflux findings score	
Infraglottic edema	0 absent
	2 present

Ventricular obliteration	2 partial
	4 complete

Erythema/hyperemia	2 only of arytenoids
	4 diffuse

Vocal fold edema	1 mild
	2 moderate
	3 severe
	4 polypoid

Diffuse laryngeal edema	1 mild
	2 moderate
	3 severe
	4 obstructive

Posterior commissure hypertrophy	1 mild
	2 moderate
	3 severe
	4 obstructive

Granuloma/granulation tissue	0 absent
	2 present

Thick endolaryngeal mucus	0 absent
	2 present

The articles were ranked according to the evidence level EBM according to the following:

1a. Systematic review articles of controlled and randomized clinical trials
1b. Controlled and randomized clinical trials
2a. Systematic review of cohort studies
2b. Cohort studies
3a. Systematic review of case–controls
3b. Case–control studies
4. Case reports
5. Specialist opinion

## Results

We found 12 articles that specifically evaluated the relationship between CRS and acid reflux in adults, with one of these being a randomized controlled trial, eight being case–control studies and three cohorts. Of these articles, eight specifically evaluated the relationship of CRS with reflux, and four articles studied the effect of treatment with PPIs (proton pump inhibitor) on the sinonasal symptoms and signs in patients with CRS and GERD.

### Studies evaluating the relation of CRS and reflux

Eight articles were found comparing pHmetry monitoring values in patients with or without CRS (Table 4).

**Table 4:** Studies evaluating CRS/reflux relationship.

Author	Type	Sample	Selection criteria	Measure	Risk of bias/EBM level	Result
Ozmen et al. (2008)^26^	Case–control	33 *vs.* 22	Waiting for nasal surgery for CRS *vs.* without CRS	Pharyngeal and esophageal pH monitoring (2 channels); middle meatus suction for analysis of nasal pepsin	Mod/2b	Reflux that is more present in the group of CRS (88%) than in controls (55%); pepsin found in most reflux patients
DelGaudio (2005)^27^	Case–control	38 *vs.* 10 *vs.* 20	CRS that is refractory to surgery *vs*. CRS solved *vs.* absence of CRS	Nasal, pharyngeal and esophageal pH monitoring (3 channels)	Mod/2b	More reflux in refractory CRS (76%) than on the other 2 groups (24%)
Ulualp et al. (1999)^28^	Case–control	18 *vs.* 34	CRS that is refractory to surgery *vs.* absence of CRS	Pharyngeal and esophageal pH monitoring (3 channels)	Mod/2b	Higher percentage of reflux in patients with CRS with laryngitis (67%) and CRS patients (33%) when compared to controls (21%)
Ulualp et al. (1999)^29^	Case–control	11 *vs.* 11	CRS that is refractory to medical treatment *vs.* absence of CRS	Pharyngeal and esophageal pH monitoring (3 channels)	Mod/2b	Higher percentage of reflux in CRS patients (64%) compared to control (18%)
Loehrl et al. (2012)^30^	Case–control	20 *vs.* 5	CRS that is refractory to medical treatment *vs.* absence of CRS	pH monitoring *via* dual-channel tube; nasopharyngeal biopsy (all patients) and lavage of nasal sinus for pepsin investigation (5 patients)	Mod/2b	LFR present in 95% of patients. Pepsin absent in nasopharyngeal biopsies but present (5/5) in lavages
Wong et al. (2004)^9^	Cohort	37	CRS that is refractory to medical treatment	pH monitoring *via* 4-channel tube	Mod/4	32.4% had GERD; LPR and reflux in nasopharynx was rare
Jecker et al. (2005)^31^	Case–control	20 *vs.* 20	CRS that is refractory to surgery *vs.* absence of CRS	pH monitoring *via* 2-channel tube	Mod/2b	GERD more present in the CRS group compared to control, but absence of LPR
Dinis and Subtil (2006)^32^	Case–control	15 *vs.* 5	CRS that is refractory to medical treatment *vs.* absence of CRS	Analysis of nasal biopsy for pepsin and *H. pylori*	High/2b	No intranasal pepsin was identified. No difference of *H. pylori* among the groups

CRS, chronic rhinosinusitis; LPR, laryngopharyngeal reflux; PPI, proton pump inhibitor; GERD, gastroesophageal reflux disease.

Ozmen et al.^26^ compared 33 patients with CRS (who had received an indication of ENS due to improvement failure after clinical treatment) to 20 patients, who would also undergo ENS for endonasal anatomical variations such as septal deformity or concha bullosa, but without CRS (confirmed by CT). pHmetry with dual-channel tube (pharynx and esophagus) was abnormal in 88% of patients with CRS and 55% of controls, being statistically significant (*p* = 0.01). This study also collected pepsin in nasal secretion during ENS; the specific activity of pepsin was detected in 82% of patients in the study group and in 50% of the control group (*p* = 0.014). In all patients with CRS, in which pepsin was detected in the nasal sample, LPR was documented by pHmetry, and only three patients with LPR at pHmetry showed negative pepsin investigation. The authors suggested that refractory CRS may be associated with LPR and that pepsin would be a good indicator for the diagnosis of LPR.

DelGaudio^27^ analyzed 38 patients with symptomatic CRS and endoscopic signs of nasal inflammation after they had been submitted to ENS, and compared them to a control group (10 patients who underwent ENS due to CRS, who remained asymptomatic after surgery, and 20 with no history of CRS or prior ENS). pHmetry was performed with three-channel tube and the author noted that the LPR was significantly more often present in the group with persistent CRS than in the control group, both when the criterion was a pH below 4 (39% *vs.* 7%) and a pH below 5 (76% *vs.* 24%) (respectively, *p* = 0.004 and *p* = 0.002). The presence of reflux in the persistent CRS group patients was significantly higher compared to the control group, both above the upper esophageal sphincter and in the distal esophagus.

Ulualp et al.^28^ evaluated several groups of patients with sinonasal complaints through with a three-channel pHmetry. The authors found a higher prevalence of acid reflux in the hypopharynx and signs of posterior laryngitis at endoscopy in patients with CRS and persistent complaints after ENS (4 of 6 patients, or 67%) when compared to healthy controls (7 of 34, or 21%) or CRS patients without posterior laryngitis (4 of 12 or 33%). There was no difference in the parameters of intensity of distal or proximal esophageal reflux between groups. He concluded that the LPR can play an important role in a subgroup of patients with CRS, and posterior laryngitis may be a common finding.

Ulualp et al.^29^ also observed a higher prevalence of LPR in a group of 11 patients with CRS, who had not responded to conventional treatment (7 of 11, or 64%) compared to 11 healthy controls (2 of 11, or 18%), in a study employing pHmetry with three-channel tube.

Loehrl et al.^30^ evaluated 20 patients with CRS with no improvement after medical and surgical treatment through pHmetry with two tubes (in the esophagus and nasopharynx), compared to pepsin in nasal secretions. The authors reported that 95% (19/20) of the patients had abnormal pHmetry in nasopharynx, and the DeMeester score values from the esophagus were changed (<14.72) in 47% (9/19) of patients. Biopsy of the nasopharynx for pepsin investigation was negative in all patients. In contrast, in five of those patients, pepsin was assessed by testing nasal lavage samples, and was positive in all cases. In five other healthy patients (with no history of paranasal sinuses diseases or GERD and negative nasal endoscopy), pepsin was not identified in nasal lavage.

Wong et al.^9^ studied 37 patients with CRS refractory to clinical treatment through pHmetry with four-channel tube that included one in the nasopharynx. The authors observed GERD in 32.4% of patients. Of the 809 episodes of reflux that were detected, using as an acid criterion a pH below 4, only 2 (0.2%) reached the nasopharynx (in two different patients). The authors concluded that reflux into the nasopharynx is a rare event and that there must be other different mechanisms of direct contact of the acid with the sinonasal mucosa for the persistence of the inflammatory process in these patients.

Jecker et al.^31^ compared a group of 20 patients with persistent CRS even after ENS to 20 healthy control patients (medical students with no history of CRS, GERD or smoking) through dual-channel pHmetry. Patients with refractory CRS had significantly more reflux events in the distal sensor (DeMeester index in patients of 32.9 ± 8.7 *vs.* controls of 6.6 ± 1.3) and the fraction with a pH below 4 was four times more frequent in patients than in controls. However, this statistical difference between the two groups was not evident with the same parameters in the hypopharyngeal sensor, which led the authors to conclude there is an association between CRS and GERD, but not with the LPR. This would support a vagal response as the most likely mechanism for this inter-relation between the two diseases.

Dinis and Subtil^32^ analyzed 15 patients with CRS refractory to clinical treatment and compared them to five controls that would be submitted to ENS due to anatomical changes (*e.g.* middle concha bullosa). He found colonization by *H. pylori* in nasal biopsies of 19% of patients with CRS; although it was present in 8% of the samples in the control group, the difference was not significant. There was no statistical difference in pepsin found in the nasal tissue, which was similar to blood levels in all patients of both groups.

Thus, most controlled studies suggest that there is a higher prevalence of reflux in a specific group of patients with refractory CRS. A limiting factor for the final conclusion is that the studies have a relatively small number of participants and are very heterogeneous in methodology, which hinders the meta-analysis.

### Longitudinal studies aimed at effect of treatment with PPIs

Four studies were found that evaluated the effect of treatment with PPI on the improvement of nasal symptoms in patients with CRS (Table 5).

**Table 5:** Longitudinal studies focusing on PPI treatment.

Author	Type	Sample	Selection criteria	Measure	Risk of bias/EBM level	Result
Vaezi et al. (2010)^33^	Controlled randomized test	75	Postnasal drip with no CRS or allergy	Symptoms evaluation and pH-metry using PPI	High/	Important improvement of symptoms compared to control group
DiBaise et al. (2002)^34^	Cohort	11	CRS that is refractory to medical and surgical treatment	Symptoms score, pH-metry *via* dual-channel tube, nasolaryngoscopy after PPI treatment for 3 months	Mod/4	Mild improvement of symptoms
Pincus et al. (2006)^35^	Cohort	30	CRS that is refractory to medical and surgical treatment	Evaluation of symptoms and pH-metry using PPI	High/4	25 of 30 had LPR or nasal reflux events. 14 of 15 improved with PPI
Durmus et al. (2010)^36^	Case–control	50 *vs.* 30	GERD and LPR *vs*. absence of GERD	Nasal saccharin test and clinical evaluation before and after treatment with PPI	Mod/2b	No difference on saccharin test. There was significant improvement of symptoms

CRS, chronic rhinosinusitis; LPR, laryngopharyngeal reflux; PPI, proton pump inhibitor; GERD, gastroesophageal reflux disease.

Vaezi et al.^33^ conducted a controlled, randomized, double-blind study to evaluate the effect of lansoprazole 30 mg twice daily in 75 patients with chronic rhinitis, a complaint of postnasal drip, no CT abnormalities in the sinuses and a negative RAST. Patients underwent pHmetry with a tube into the esophagus and impedance monitoring before treatment, and were followed by validated questionnaires (SNOT-20, RSOM-31 and QOLRAD) 8 and 16 weeks after initiation of treatment. Patients receiving therapy with lansoprazole were 3.12 times (at 8 weeks of treatment) and 3.5 times (after 16 weeks of treatment) more likely to notice improvement of their postnasal drip compared to controls. After 16 weeks, the average improvement in the treatment arm was 50% compared to 5% in the placebo group. There was also a significant improvement in the SNOT-20 and QOLRAD scores in the treatment arm. The presence of reflux in pHmetry before treatment was not decisive for the answer.

In a prospective study, DiBaise et al.,^34^ compared 11 patients who had failed clinical and surgical treatment of CRS to 19 patients with GERD who had no CRS (no nasal symptoms and negative nasal endoscopy), evaluating sinonasal symptoms and reflux with a non-validated questionnaire (that assessed 14 symptoms of GERD and rhinosinusitis, the frequency of these symptoms, improvement with treatment, and overall satisfaction) and with dual-channel pHmetry. A similar percentage of abnormal pHmetry was observed between the two groups (82% in the CRS group and 79% in the GERD group) at baseline. Treatment with 20 mg omeprazole twice daily for 12 weeks was instituted only in the CRS group, and this was reassessed on a monthly basis. The authors noted modest improvement in symptoms and overall satisfaction with the treatment among these patients.

Pincus et al.^35^ performed pHmetry in 30 patients with CRS with no improvement with clinical and surgical treatment. Of these, 25 had an associated diagnosis of GERD. For these patients, a treatment with PPIs was started, with an interview being performed by telephone one month later. Of the 15 patients who were re-evaluated, 14 reported improvement of nasal symptoms, and seven fully improved their complaints.

Durmus et al.^36^ studied 50 patients with GERD and LPR, based on clinical and endoscopic diagnosis, and compared them to 30 healthy patients. Pretreatment tests of sucrose were similar between the two groups, while the RSI and RFS questionnaires were significantly worse in the group of LPR. All patients then underwent a treatment with lansoprazole 30 mg twice daily for 12 weeks. There was no statistical difference between the results of saccharin test in the control and study groups before treatment. After treatment, the differences between RSI and RFS remained similar to pre-treatment levels, as did the saccharin test results. These authors concluded that both GERD and the LPR do not seem to affect the nasal mucociliary transport.

Thus, current studies available in the literature are conflicting as to the effect of PPI therapy in symptom improvement in patients with CRS. In addition, CRS or reflux diagnoses were not confirmed by complementary tests, which makes the real interpretation of the results difficult.

## Discussion

LPR was present with significantly greater difference in patients with CRS compared to groups of patients without CRS in 4 studies.^26^, ^27^, ^28^, ^29^ Although they were controlled studies, none of the groups were matched for age, weight, anatomical abnormalities predisposing to reflux (such as hiatal hernia), previous treatment of GERD, or findings of upper digestive endoscopy. In addition, there is great variability in the use of pHmetry for diagnostic confirmation, with respect to the number of tubes, their positioning, and criteria used for diagnosis confirmation. This huge variability makes definitive conclusion on the subject even more difficult.

Two studies did not show a relationship between high acid reflux (LPR or nasopharynx) and CRS.^9^, ^31^ In one such study (Jecker et al.),^31^ despite similar LPR findings between the control group and study group, the group with persistent CRS after ENS had a higher prevalence of GERD than the control group. These authors even suggested that there should be an association between the two diseases, probably mediated by vagal reflex.

Some studies used an analysis of pepsin in the nasal cavity for diagnosis of reflux. While Loehrl et al.^30^ and Dinis and Subtil^32^ analyzed a nasal tissue biopsy and did not observe the presence of pepsin in their results, the pepsin in nasal lavage was present in large amounts in patients with CRS in two studies.^26^, ^30^ The comparison of pepsin in lavage with the control group, however, was not consistent: while Loehrl et al.^30^ observed an amount that is significantly greater in the CSR group compared to the control, Ozmen et al.^26^ reported that the control group also showed high amount of pepsin, and that there was no significant difference between the groups analyzed. Apparently the result of this test depends greatly on the collection technique, with sensitivity being higher when pepsin is collected in nasal lavage than when it is evaluated in a nasal biopsy. Moreover, the small number of subjects in each of the studies makes the final analysis on the topic impossible. Finally, we found no data in the literature to validate the collection of pepsin in the nasal cavity as a test to be used for reflux investigation.

In general, the studies currently available suggest that there is a relationship between reflux and a specific subtype of CRS, refractory to clinical and surgical treatment. However, studies are few, and the small number of patients and the different methodologies, employed make it difficult to conduct a meta-analysis. All these hinder the most reliable interpretation of the data. Thus, more controlled studies with larger numbers of patients and probably multicenter participation will be needed to confirm this hypothesis.

When randomized controlled trials were conducted to assess the improvement of symptoms of CRS after treatment of reflux, Vaezi et al.^33^ observed improvement of postnasal drip in the evaluated patients, with no CRS or allergies. Pincus et al.^35^ reported a significant improvement of symptoms of CRS after treatment, while DiBaise et al.^34^ reported that treatment with PPIs showed a slight impact on the improvement of symptoms. Durmus et al.^36^ reported that there was no difference in pre- and post-treatment saccharine test with PPIs for three weeks, although they noted improvement in reflux symptoms. Thus, current controlled, randomized, double-blind studies available in the literature, describe extremely different methodologies. Even worse, many fail to confirm the diagnosis of CRS or GERD/LPR and are based only on the improvement of nasal symptoms. Thus, multicenter studies, with a more significant number of patients, that have specified criteria for diagnosis, and standardized methodology, should help considerably in elucidating this question.

## Conclusions

According to the studies found in the literature, it was concluded that there appears to be relative prevalence of reflux in patients with difficult to control CRS. However, controlled studies with a significant number of patients are lacking to confirm this hypothesis. Similarly, there are few studies that specifically assess the impact of treatment of reflux in symptom improvement in patients with CRS.

## Conflicts of interest

The authors declare no conflicts of interest.
